# Herpesvirus Infection of Endothelial Cells as a Systemic Pathological Axis in Myalgic Encephalomyelitis/Chronic Fatigue Syndrome

**DOI:** 10.3390/v16040572

**Published:** 2024-04-08

**Authors:** Jean M. Nunes, Douglas B. Kell, Etheresia Pretorius

**Affiliations:** 1 Department of Physiological Sciences, Faculty of Science, Stellenbosch University, Stellenbosch, Private Bag X1, Matieland 7602, South Africa; mass.nunes@gmail.com; 2 Department of Biochemistry, Cell and Systems Biology, Institute of Systems, Molecular and Integrative Biology, Faculty of Health and Life Sciences, University of Liverpool, Crown Street, Liverpool L69 7ZB, UK; 3 The Novo Nordisk Foundation Center for Biosustainability, Technical University of Denmark, Building 220, Chemitorvet 200, 2800 Kongens Lyngby, Denmark

**Keywords:** myalgic encephalomyelitis/chronic fatigue syndrome (ME/CFS), endothelial cells, herpesvirus

## Abstract

Understanding the pathophysiology of myalgic encephalomyelitis/chronic fatigue syndrome (ME/CFS) is critical for advancing treatment options. This review explores the novel hypothesis that a herpesvirus infection of endothelial cells (ECs) may underlie ME/CFS symptomatology. We review evidence linking herpesviruses to persistent EC infection and the implications for endothelial dysfunction, encompassing blood flow regulation, coagulation, and cognitive impairment—symptoms consistent with ME/CFS and Long COVID. This paper provides a synthesis of current research on herpesvirus latency and reactivation, detailing the impact on ECs and subsequent systemic complications, including latent modulation and long-term maladaptation. We suggest that the chronicity of ME/CFS symptoms and the multisystemic nature of the disease may be partly attributable to herpesvirus-induced endothelial maladaptation. Our conclusions underscore the necessity for further investigation into the prevalence and load of herpesvirus infection within the ECs of ME/CFS patients. This review offers conceptual advances by proposing an endothelial infection model as a systemic mechanism contributing to ME/CFS, steering future research toward potentially unexplored avenues in understanding and treating this complex syndrome.

## 1. Introduction

Myalgic encephalomyelitis/chronic fatigue syndrome (ME/CFS) is a chronic condition characterized by unresolved fatigue, cognitive dysfunction, malaise, orthostatic issues, and post-exertional symptom exacerbation (PESE), among other symptoms [1]. The etiological cause is officially unknown, but viral infection is believed to be a precipitating factor, and its pathology is very much associated with viral activity [2,3,4,5].

Herpesviruses are the most implicated in ME/CFS research [2,4,5,6,7,8,9,10], and as such, the relationship between herpesviruses and ME/CFS has been reviewed extensively [2,4,10,11,12]. However, because the majority of the global population is infected with herpesviruses, elucidating a mechanistic role for these viruses in ME/CFS is a difficult undertaking. Herpesviruses have the ability to infect a number of different cell types within the body, but they exhibit a preference for a particular population. For instance, the primary target cells of EBV are B-cells [13], whereas human cytomegalovirus (HCMV) attacks non-lymphoid cells, of which endothelial cells are a favoured cell type for infection [14,15]. That is not to say, however, that EBV, for example, is unable to infect cell types other than B-cells and cause significant pathological consequences.

Endothelial dysfunction is another prominent characteristic of ME/CFS pathology and has been repeatedly demonstrated in both older and more recent studies [16,17,18,19,20,21,22,23,24]. Blood flow, especially cerebral blood flow, is perturbed and reduced in ME/CFS patients [25,26,27,28,29,30,31], as well as the perfusion of various brain regions [32,33,34,35]. ME/CFS is not typically viewed as a vascular disease, but the aforementioned findings as well as evidence of endothelial and vascular dysfunction in Long COVID [19,36,37,38], a disease that shares many symptoms with ME/CFS [39,40,41,42], begs the question as to whether or not endothelial and vascular pathology are important factors for ME/CFS pathology and symptom manifestation.

We have recently demonstrated haematological pathology in ME/CFS platelet-poor plasma (PPP) samples, specifically pertaining to platelet and clotting processes [43]. We found significant levels of amyloid fibrin(ogen)/microclots—the clotting material that is implicated in Long COVID [38,44,45]—in ME/CFS PPP samples. These microclots have been therapeutically targeted with considerable success in Long COVID patients [46]. Other research groups have also demonstrated platelet abnormalities in ME/CFS cohorts [47,48,49], as well as abnormalities in clot formation and kinetics [50]. It is well known that the integrity of endothelial cells and their normal signalling are paramount factors for the regulation of coagulation [51,52], and this leads to the recognition that endothelial dysfunction might be, at least in part, responsible for the abnormalities in coagulation and platelet function observed in certain ME/CFS patients.

The ideas of impaired circulatory function, reduced tissue oxygen supply, and unmet metabolic demands revolving around endothelial dysfunction and its inability to correctly regulate vascular tone have been discussed in the context of ME/CFS pathology and symptom manifestation before, and have even been tied to symptoms such as fatigue and cognitive dysfunction [53,54,55,56].

**Figure 1:**
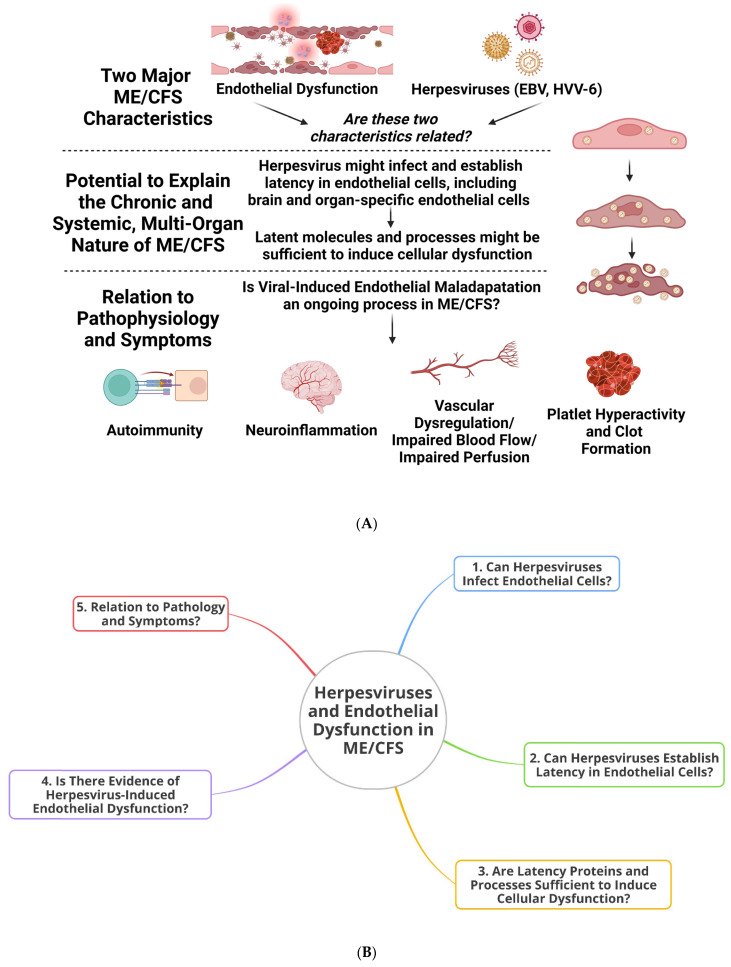
An overview of the present paper. (**A**) A delineation of our theoretical approach and (**B**) a chronological mind map (created at https://app.ayoa.com/mindmaps (accessed on 21 November 2023)).

Circling back to viruses, very few or no studies have focussed on herpesvirus infection of the endothelium in ME/CFS or the consequences that this might have for pathology and symptom manifestation. It is acknowledged that herpesviruses can induce pathology independent of the endothelium, which is a phenomenon that certainly has relevance to ME/CFS and other diseases. However, here, we aim to focus on herpesviruses and the endothelium or associated tissues. There are a number of different herpesviruses, but the focus here is on the ones significantly implicated in ME/CFS, namely HHV-4 (EBV) and HHV-6 [2,3,4,5,57,58]. For an overview of the ideas presented in this paper, as well as a mind map for clarification, see Figure 1.

## 2. Infection of Endothelial Cells by Herpesviruses, Latent Modulation, Systemic Complications, and the Potential for Long-Term Maladaptation 

With relevance to the present idea, endothelial cells (ECs) are able to be infected by EBV [59,60,61,62,63,64,65,66] and HHV-6 [67,68,69,70,71,72]. Other herpesviruses, including HHV-8 and HCMV, also infect ECs [73,74,75,76,77,78]. Since herpesviruses are intimately associated with ME/CFS, this already provides reason to hypothesize that the endothelium in ME/CFS patient is, to some extent, infected by herpesviruses.

Next, it is important to discuss evidence that suggests or indicates that herpesvirus latency, specifically, can occur in ECs. Herpesviruses establish lifelong infection/latency by either integrating their genome into the host genome or, more commonly, having the genetic material exist in the nucleus as an episome [79]. Differences in latency between herpesviruses can be found elsewhere [80]. With regard to HHV-6, where some studies have inferred that ECs act as reservoirs for HHV-6, where low-level replication ensues [70,71], there are studies that have specifically demonstrated that HHV-6 establishes latency in ECs [81,82]. Moving onto EBV, there is a lack of studies that demonstrate that this herpesvirus can establish latent infection in ECs. The latent infection of brain microvascular ECs and the reactivation thereof have been suggested to play important roles in multiple sclerosis [65], but the exact mechanisms around EBV latency in these cell types seem to remain unestablished. Certainly, further research is required in the context of these herpesviruses and EC latency, but the evidence at hand supports the writing of this paper’s hypothesis in the meantime. 

Active viral infection has been shown in ME/CFS [3,57,83,84], but has not been specifically studied in an endothelial context. Active infection might not necessarily coincide with all symptoms, as active infection is not necessarily a continuous process; hence, active infection might fall short in providing a unitary explanation for daily symptoms. While active infection will certainly involve the endothelium, it may be true that latency is sufficient enough to induce endothelial dysfunction that brings about ME/CFS symptoms—that is, if herpesvirus latency occurs within ECs, it is possible that latency in non-ECs can indirectly cause endothelial dysfunction too, as discussed later. 

As with the development of bacterial dormancy [85,86], herpesvirus latency is not a passive process [79,87,88], especially from the perspective of the host cell. There are viral proteins and nucleic material that regulate latency and reactivation, modulate host cell functions and proliferation, and ensure viral subversion of the immune system [79,89]. The activity of herpesvirus latency and all the specific molecular processes that occur within the host nucleus (as well as those that occur in the cytosol and extracellularly) might bring about specific defects at the endothelial layer. Immune and neurological systems are certainly involved, but so might the endothelium. 

ME/CFS is a chronic condition, and the chronicity of symptoms bears an explanation. A herpesvirus infection of ECs might be able to explain the persisting nature of ME/CFS symptoms. Firstly, the endothelium has a low turnover, with the entire population being replaced every 6 years in adults [90]. Other studies estimate that this occurs anywhere from months to decades [91], but note that tissue-specific ECs vary in their rates of turnover. Regardless, the idea is that the endothelium is a normally long-lasting tissue, and viral-induced dysfunction caused by persisting viruses that can establish latency, may continue for the rest of an EC’s life. Relevantly, EBV inhibits apoptosis in ECs [92] and can increase the persistence of dysfunctional ECs. HHV-6 also inhibits apoptosis of host cells [93].

This ability of herpesviruses to establish latency in (endothelial) host cells, along with the long life span of ECs, means that a herpesvirus-infected endothelium and the resulting complications may persist for months to years. This is a timeline that is in accord with the chronicity of ME/CFS symptoms. Hence, it is plausible that ME/CFS ECs undergo chronic maladaptation as a result of (latent) herpesvirus infection, which might be important for the maintenance of pathology and symptom manifestation. Furthermore, this may be an ongoing phenomenon in other diseases too, such as Long COVID. 

A particular significance of this reasoning is its focus on the cell type that we are postulating to be involved and infected. ECs are the interface between blood and tissue. They enable gas exchange, nourishment, and waste clearance, regulate inflammatory processes, secrete bioactive amines that contribute to haematological and vascular homeostasis, and perform many other essential physiological functions. Any dysfunction in these cells and their functional processes are likely to contribute to or induce pathology. Furthermore, the vasculature extends into every organ system, and hence, such endothelial dysfunction might account for the multi-organ and systemic nature of ME/CFS pathology. 

## 3. Latent Infection by Herpesviruses Is Sufficient to Bring about Cellular Dysfunction and Might Hold Relevance for Endothelial Dysfunction and Symptom Manifestation in ME/CFS

Herpesviruses are persistent viruses that affect host cells for a lifetime, supporting the notion that latency might be able to cause chronic pathologies like ME/CFS in susceptible individuals. It is emphasized that persistent, latent infection is not a passive process and in fact exerts pathological effects on the host [87,88,94,95]. Hence, herpesvirus reactivation and active infection may not be necessary for the manifestation of ME/CFS symptoms, although they are expected to exacerbate any issues. Here, we aim to show that latent proteins from herpesviruses are sufficient to induce cellular dysfunction and hint at the idea that they and latency-related processes contribute to the endothelial and vascular dysfunction—as well as other pathophysiological characteristics—observed in ME/CFS. 

Herpesviruses use a number of proteins and microRNAs to drive latency, evade immune surveillance, regulate host processes, and coordinate the transition to active infection [79,96]. EBV is a heavily studied herpesvirus, most notably due to its ability to transform lymphocytes and epithelial cells into malignant phenotypes [97,98], and for its role in causing infectious mononucleosis. In fact, it is well known that EBV latent genes and their products specifically interrupt cell cycles and promote oncogenesis [96], and both latent and lytic gene products illicit notable immune responses [99]. Epstein–Barr Nuclear Antigens (EBNAs) are a group of proteins encoded by EBV that are essential for viral genome replication and transcription, the establishment and regulation of latency (even though some function in lytic processes), and immune evasion [100,101,102]. Other latency-associated molecules encoded by EBV include latent membrane proteins (LMPs) and EBV-encoded small RNAs (EBERs) [103,104]. 

EBNA-1 significantly increases ROS production in the host cells that they infect, and, through this mechanism, contributes to DNA damage and the inhibition of the repair thereof [105,106,107]. It inhibits apoptosis and enhances cell survival, contributing to its recognition as an oncoprotein [108]. The expression of this latent protein in ECs is associated with higher IL-6 production [64] and hints at the potential of an EBV-infected endothelium to adopt a proinflammatory phenotype with a range of downstream consequences, including immune activation, increased clotting propensity, and vascular dysregulation. Immune responses against EBNA-1 also lead to the production of autoantibodies [109,110], which might have relevance to autoimmunity in ME/CFS [53,111,112]. EBNA-1 is also associated with upregulations of IL-8, hypoxia-inducible factor-1 alpha, and vascular endothelial growth factor (VEGF) [113], of which the latter two promote angiogenesis and influence vascular integrity and dynamics. 

EBV’s LMP-1, through NF-κB, increases the expressions of cyclooxygenase (COX) 2, prostaglandin E_2_, and VEGF [114]. In fact, LMP-1 is capable of activating several forms of NF-κB, involving a number of different signalling mechanisms [115], and it also activates JAK3, p38, mitogen-activated protein kinases, and several STAT-related proteins [116,117]. Importantly, in ECs, LMP-1 leads to NF-κB activation and increased expressions of IL-1β, IL-6, IL-8, monocyte chemotactic protein-1, RANTES, ICAM-1, VCAM-1, and E-selectin, as well as the inhibition of caspase-3 and hence a reduction in apoptotic tendencies [92]. This study emphasizes the potential cellular dysregulation that occurs in ECs as a result of herpesvirus latency, as well as localized and potentially systemic physiological disturbances, including the activation and binding of immune cells and platelets to the endothelium. 

Endocan is upregulated by LMP-1, again through NF-κB, and levels of endocan and LMP-1 have been positively correlated in patient tissue samples [118]. Endocan can promote endothelial dysfunction and cardiovascular disease by increasing inflammation, oxidative stress, and the expression of adhesion molecules [119]. LMP-1 increases the sumoylation of proteins related to cellular migration and transcriptional activity [120] and also increases glycolytic processes and interferes with the host metabolism [121,122,123]. LMP-1 also modulates host epigenetic processes [124,125,126] and hinders DNA repair mechanisms [127]. 

**Figure 2:**
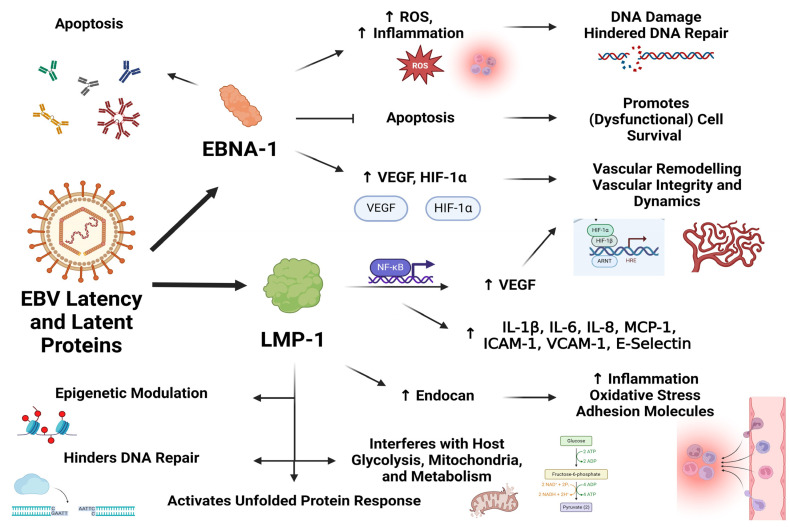
Some mechanisms through which EBV latent proteins EBNA-1 and LMP-1 can induce cellular dysfunction in endothelial cells.

Host protein synthesis is inhibited, and autophagy and the regulation thereof are interrupted by LMP-1 [128], and this latency protein also activates the unfolded protein response [129]. Lastly, LMP-1 also interferes with mitochondrial regulation and cell metabolism by altering the phosphorylation of the mitochondrial dynamin-related protein 1 [130]. LMP-2A exerts potent anti-apoptotic effects and aids in immune evasion by reducing the reactivity of CD8+ T cells to cells infected by EBV [131,132]. RNAs from EBV, EBERs, are also associated with cellular dysfunction and proinflammatory processes [133] and represent other mechanisms through which EBV latency can bring about cellular dysfunction. Figure 2 represents the mechanisms discussed through which EBV latency proteins EBNA-1 and LMP-1 can induce cellular dysfunction in endothelial cells.

In ECs, U94, a latency-associated protein from HHV-6 [134], reduces cell migration and angiogenic potential (by desensitizing the response to VEGF) and thus leads to prolonged wound healing [81,135]. U94 increases the expression of human leukocyte antigen G, which is believed to underlie its effects on angiogenesis [68]. This latent protein from HHV-6 also shows anticancer potential as it inhibits DNA repair genes and aspects of the cell cycle and leads to apoptosis through the intrinsic pathway [136]. While U94 shows potential in cancer therapy, its effects on DNA repair and cell function might not be so favourable in non-malignant, healthy cells. 

Ultimately, latency-related molecules and processes are sufficient to induce (endothelial) cellular dysfunction. The latency of herpesviruses, especially when carried out in a particular cell type (such as ECs), might have consequences for systemic physiology. The extent to which this is an ongoing process in ME/CFS warrants further investigation.

## 4. Evidence for Herpesvirus-Induced Endothelial Dysfunction

We next present the links between herpesvirus infection and endothelial pathology specifically (Table 1). This section focuses on direct and indirect mechanisms but is not exclusive to latency-related molecules and processes. 

## 5. Herpesvirus-Induced Endothelial Dysfunction and Its Relevance to ME/CFS

We have discussed the potential of herpesviruses to infect and establish latency in ECs, how their latent proteins and processes are sufficient to induce (endothelial) cell dysfunction, and some of the evidence of herpesvirus-induced endothelial dysfunction. Now, we want to touch on some of the pathophysiological characteristics of ME/CFS and how they might relate to the present discussion thus far. 

## 6. Endothelial Cells, Vascular Dysregulation, and Perfusion: Do Herpesviruses Have a Role to Play in the Dysregulation of Blood Flow Observed in ME/CFS?

One of the most important findings in ME/CFS research is that of reduced cerebral blood flow in patients, even in those without tachycardia and hypotension [25,26,28,29]. The orthostatic symptoms associated with ME/CFS are not due to deconditioning [172,173], suggesting an underlying defect in blood flow regulation, perhaps related to autonomic dysfunction [174]. viral infection of vascular cells, such as ECs and smooth muscle cells, and neurons might contribute to the blood flow and perfusion abnormalities of ME/CFS. 

As we have seen, herpesviruses can significantly affect ECs and result in structural and functional changes, which have consequences for the physiological roles of ECs. Endothelial dysfunction is associated with and contributes to impaired tissue perfusion [175,176,177,178,179], and there is even evidence demonstrating an association between herpesviruses and the impaired perfusion of tissues [66,138,180]. 

**Figure 3:**
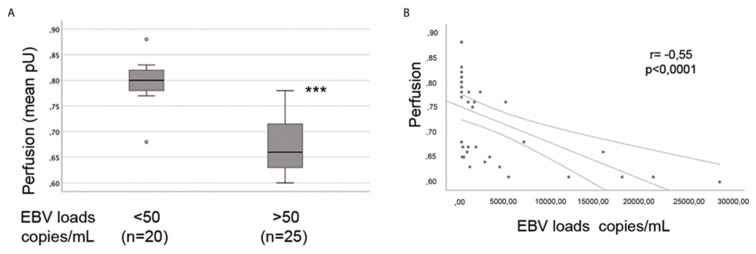
(**A**,**B**) Adopted figure from [66] showing that the EBV load is inversely associated with hand skin perfusion (Open Access). (*** = *p* > 0.001).

EBV and HHV-6 are associated with reduced cerebral blood flow and the perfusion of particular regions [180,181,182]. Furthermore, Farina et al., (2021) demonstrated that skin perfusion is significantly reduced in patients with higher EBV loads in the blood compared to patients with low or undetectable viral loads (hence, the EBV load is inversely associated with blood perfusion; refer to the adopted Figure 3). 

HHV-6 encephalopathy is associated with reductions in cerebral blood flow and the perfusion of the frontal lobe [181], as well as perturbations in coronary microcirculation [151]. Similarly, EBV encephalitis is also associated with reduced cerebral blood flow [180]. Caruso et al. (2002) showed that large-vessel ECs, specifically aortic ECs are more susceptible to infection by HHV-6 than are ECs of the microvasculature [67], and might have relevance to the reduced cerebral blood flow in ME/CFS.

Viruses (and bacterial LPSs) can damage the glycocalyx of the endothelial layer [37,183,184,185,186]. A damaged glycocalyx impairs perfusion and also increases the risk of mortality in hospitalised patients [179,187,188,189,190,191]. As mentioned earlier, EBV can also damage endothelial cellular junctions and hence endothelial barriers [144]. HHV-6 can cause fibrosis in ECs [192] and may have implications for perfusion and the ability to exchange substances across vessel walls. These are possible mechanisms through which herpesviruses might bring about impairments in vessel regulation, blood flow, and perfusion. 

Long COVID, a disease extremely similar to ME/CFS, is initiated by SARS-CoV-2 in a minority of patients (which ranges from 10–30%) who are acutely infected. In a histopathological examination of penile tissue from two males with and two males without a history of COVID-19 infection, viral RNA was detected, and the virus particles were found in the proximity of ECs in the COVID-positive patients [193]. Furthermore, the eNOS expression was also decreased in the COVID-positive samples. The researchers inferred that systemic (and of course localized) COVID-19-induced endothelial dysfunction can result in erectile dysfunction. If this is the case, then it emphasizes the extent to which virus-infected/affected ECs can impair tissue perfusion.

Hence, the infection of ECs and associated cells through herpesviruses might play contributory roles in the blood flow deficits and vascular dysregulation observed in ME/CFS [25,26,27,28,29]. An investigation of vascular tissue from patients can further inform our understanding of vascular dysfunction and impaired tissue perfusion in ME/CFS. Furthermore, herpesviruses might also contribute to this issue by infecting neurons and causing the dysregulation of autonomic control [53,54,174]. 

## 7. Herpesviruses, Endothelial Cells, Platelets, and Coagulation

Related to the platelet abnormalities of a procoagulant phenotype found in ME/CFS patients [43,47,48,49,50], there have been cases where EBV infection caused/was associated with severe cardiac and vascular issues, including myocarditis, vasculitis, disseminated intravascular coagulation, venous thromboembolism, thrombotic thrombocytopenic purpura, deep vein thrombosis, and stroke [194,195,196,197,198,199,200,201,202], emphasizing EBV’s role in haematological and vascular pathologies. An EBV infection of ECs causes a significant increase in the von-Willebrand factor (VWF), VEGF, and platelet endothelial cell adhesion molecule-1 (PECAM) levels [143], which contribute to procoagulant processes. ECs participate in coagulation and the regulation thereof, and hence minor cellular disturbances, such as an increase in the endothelial vWF expression caused by EBV [143], will have significant effects on clotting processes.

It has also been shown that EBV can affect the function of platelets by modulating their mRNA and non-coding RNA profiles [203]. Whether or not this leads to platelet hyperactivation and subsequent coagulant processes was not assessed in the previous study. There is also evidence that suggests an association between autoantibodies developed against platelet antigens and active EBV infection [204,205]. However, the previous mechanism is believed to contribute to thrombocytopenia, and hence a reduced prothrombotic state from the platelet perspective. To add, an infection of lymphocytes by EBV can also induce a procoagulant state. It has been shown that the supernatant from EBV-infected NK cells lead to increased procoagulant when exposed to monocytes, particularly by upregulating the expression of the tissue factor [206]. 

HHV-6 infection is associated with prothrombotic states [83,149]. Specifically, it is associated with thrombotic microangiopathy [207]—whether this is related to fibrinaloid microclots, which are found in both Long COVID and ME/CFS cohorts, requires further investigation [43,44]. To note, a prothrombotic state associated with active HHV-6 in an ME/CFS cohort has been reported [83], albeit in an old study. HHV-6 reactivation is also accompanied by an increase in plasminogen-activator 1 [148], which leads to reduced clot lysis and clearance.

Hence, there is reason to suspect that the clotting and platelet abnormalities noted in ME/CFS [43,47,48] arise from the consequences of herpesvirus-infected ECs, as well as other procoagulant effects of herpesviruses independent of endothelial cells. 

## 8. Herpesviruses and Neurological Issues in ME/CFS: Implications at the Cerebro-Endothelium? 

Endothelial dysfunction has been associated with cognitive dysfunction in vascular dementia [208] coronary artery disease [209], obesity [210], postoperative cognitive dysfunction [211], type II diabetes [212], sleep apnoea [213], and the elderly [214]. Endothelial markers, such as endothelial lipase, positively correlate with cognitive impairment [215]. A systematic review inferred an ‘intrinsic’ relationship between endothelial dysfunction and vascular cognitive impairments [216]. These studies suggest that endothelial dysfunction might have a significant role to play in the neurological issues suffered by ME/CFS patients. 

As we have discussed in this paper, many of the herpesviruses are capable of infecting brain microvascular ECs, which might act as viral reservoirs from which CNS infection can ensue. Furthermore, a compromised BBB is an expected consequence. Importantly, EBV and HHV-6 have been detected in CNS tissue from deceased ME/CFS patients [5]—cerebrovascular ECs might be the reservoir/latent site for these viruses in patients. EBV infection is associated with cognitive impairments [217,218], and EBV proteins, including EBV dUTPase, are posited to contribute neuroinflammation and subsequent neurological issues in ME/CFS [9]. HHV-6 is also associated with cognitive impairments [219,220,221,222]. HHV-6 antigens have been found within ECs from the frontal lobe of a fatal case of herpesvirus infection [161], and in a mice study, HHV-6 infection of the CNS persisted and induced proinflammatory cytokine production through TLR-9 [169]. 

The infection of brain ECs and other vascular cells (as well as neurons and glial cells) by herpesviruses and subsequent vasculitis in brain blood vessels might lead to inflammation, CNS infection, oxidative and NS damage, and symptom expression including cognitive dysfunction and even fatigue and PESE. Neurological pathology might also ensue as a result of autoimmune processes in the central nervous system, whereby molecular mimicry involving EBV and other herpesvirus antigens result in autoantibodies directed against brain antigens, for example, the glial cell adhesion molecule [110]. This may have relevance to ME/CFS and tie together working hypotheses on endothelial dysfunction and neurological symptoms in ME/CFS [55] with herpesvirus activity. 

## 9. Ways Forward

It is of interest to determine whether ECs from patients suffering from ME/CFS (and controls) are infected with herpesviruses. This would be the first step in confirming the idea presented in this paper. ECs, and brain microvascular ECs specifically, can be extracted and isolated using a number of techniques [223,224,225,226]. Other vascular tissue cells, such as smooth muscle cells, should also be isolated and tested for herpesvirus infection. Whilst there have been studies that have demonstrated a reduced permeability of endothelial linings as a result of herpesvirus infection and studies associating herpesvirus infection with perfusion deficits, further experiments focussing on these phenomena and the molecular processes involved, from a post-viral perspective, are necessary. 

## 10. Conclusions

We presented the idea that a herpesvirus infection of ECs might be an important, overlooked phenomenon that can, in part, account for the pathophysiology and symptoms of ME/CFS and, potentially, Long COVID. We do acknowledge that ME/CFS is an extremely heterogeneous disease with numerous proposed etiological factors, with various subpopulations experiencing specific symptoms and presenting a clinically distinct phenotype. Hence, this idea may not explain the symptoms of all ME/CFS subpopulations—further work is required in this context. Additionally, there exist pathogens other than herpesviruses that can influence endothelial function and lead to downstream effects, and hence, this idea is not entirely exclusive to herpesviruses.

This idea is somewhat novel but is not unprecedented [84,227,228,229,230]. Endothelial dysfunction and herpesvirus activity are two characteristics of ME/CFS pathology that have yet to be officially linked. Perhaps the latent infection of ECs alone (that is, without active infection) is sufficient to bring about pathology and subsequent symptoms and may provide an explanation for daily symptoms, the chronicity of symptoms, and the multi-organ, systemic nature of ME/CFS. 

Importantly, the load of EC infection, i.e., how widespread the systemic vasculature herpesvirus infection is, and perhaps the tissue- and organ-specific site of infection are likely vital factors that determine the manifestation of symptoms as a result of this hypothesized pathophysiological process. It is acknowledged that herpesviruses infect many cell types, so the infection of ECs by herpesviruses is likely not responsible for all ME/CFS pathologies and symptoms. For example, the infection of immune cells and manipulation of immune processes by herpesviruses contribute to ME/CFS pathology, and likely account for much of the immune disturbances seen in this population; similarly, infection of cells of the nervous system might account for neurological deficits and even vascular dysregulation. However, we want to bring attention to the possibility of endothelial infection, as this is somewhat of an understudied topic, especially in the context of ME/CFS.

Further studies are required to determine the extent of a herpesvirus infection of the endothelium in ME/CFS patients, and these need to take into account the possibility of tissue- or organ-specific sites of infection. This is a difficult phenomenon to prove, especially when considering the ability of herpesviruses to cause pathology in certain individuals and in certain physiological states, hence requiring diligent and elaborative study and experimentation. It is possible that this idea of herpesvirus latency-induced endothelial maladaptation might turn out to be irrelevant, but herpesvirus-induced endothelial dysfunction, with and without a direct infection of ECs, will still be relevant for ME/CFS pathology and symptom manifestation. Hence, a more refined focus on herpesviruses and endothelial function and health in ME/CFS (and Long COVID) is warranted. 

## Figures and Tables

**Table 1:** Links between herpesvirus infection and cellular dysfunction in ECs.

Links between Herpesvirus Infection and Endothelial Dysfunction	References
EBV	
ECs infected with EBV exhibit a proinflammatory phenotype, along with NF-κB and TLR9 activation, increased interferon, cytokine, and adhesion molecule expressions, and increased clotting propensity	[66,92,137]
ECs increase the expressions of markers associated with vascular injury, such as endothelin-1, thrombospondin 1, and heparan sulphate proteoglycan 2	[66]
Monocytes have the ability to transfer EBV infection to ECs	[138]
Microvascular brain ECs infected by EBV exhibit a proinflammatory phenotype and lead to leukocyte recruitment	[65,139]
The upregulation of endothelial adhesion marker VCAM-1 upon infection	[140]
EBV-infected macrophages induce proinflammatory sequelae in ECs and increase adhesion molecule expression	[141]
EBV dUTPase compromises the blood–brain barrier integrity	[9]
EBV alters cholesterol, polysaccharide, nucleotides, nucleic acid, and proline moieties in infected brain microvascular ECs	[137]
EBV-infected ECs of genital origin express LMP-1 on their membranes	[142]
The endothelial microenvironment is influenced by EBV infection	[143]
Extracellular vesicles from EBV-infected cells damage endothelial gap junctions and prompt endothelial-to-mesenchymal transitions	[144]
The modulation of host autophagy in endothelial cells	[145]
Exosomes containing EBV-proteins can cross brain ECs and enter the central nervous system	[146]
EBV protein-containing exosomes can lead to long-term endothelial dysfunction	[147]
HHV-6	
HHV-6 infection is associated with endothelial dysfunction and a greater extent of endothelial damage than HCMV	[81,148,149]
HHV-6 infects ECs but does not induce cytolytic effects, which led to the conclusion that ECs act as a reservoir for HHV-6 in vivo	[67]
HVV-6 is able to maintain a low level of replication within ECs	[70,150]
An association between HHV-6 and endothelial dysfunction coupled to microcirculatory defects was demonstrated	[151]
The induction of endothelial dysfunction by HHV-6 and subsequent influence on perfusion were alluded to	[152]
HHV-6 antigens, DNA, and virus particles were found in ECs and associated vascular tissue from patients suffering from various cardiovascular diseases	[72,153,154,155,156,157]
Cardiac dysfunction, specifically reduced LVEF, is associated with HHV-6 DNA persistence in endomyocardial biopsies and is ameliorated when HHV-6 latency is resolved	[158]
Considered to be a major cause of viral myocarditis	[159]
HHV-6 also infects the CNS and ECs lining its vasculature	[160,161,162]
HVV-6 is implicated in neurological disease	[163,164,165,166,167,168]
Much like EBV, HHV-6 uses TLR9 to upregulate inflammation and promote lymphocyte filtration, as was revealed in a study where mice infected with HHV-6 subtypes resulted in CNS infection and viral persistence in brain tissue for up to 9 months	[169]
HHV-6 induces cellular inflammation and upregulates the expressions of IL-8, RANTES, and monocyte chemoattractant protein-1 in ECs, even in a latent state, without viral DNA replication	[67,69,170]
It can also promote the reactivation of EBV	[171]
Lymphatic ECs also succumb to latent infection by HHV-6, where EC angiogenic and migratory properties are modulated	[81]
